# Portocath insertion technique: retrospective study & step-by-step surgical description without tunneling in a high-complexity service

**DOI:** 10.1590/0100-6991e-20223167

**Published:** 2022-03-11

**Authors:** ANNA MARIA GARCIA CARDOSO, FERNANDA SANTOS WENGROVER, ALINE WÜRZIUS, MARINA PUERARI PIETA, RAFFAELA NASCIMENTO DE CARLI, CARLOS EDUARDO BASTIAN DA-CUNHA, RICARDO BREIGEIRON

**Affiliations:** 1 - Hospital da Pontifícia Universidade Católica do Rio Grande do Sul - PUCRS, Cirurgia Geral - Porto Alegre - RS - Brasil; 2 - Universidade de Ciências da Saúde de Porto Alegre - UFCSPA, Escola de Medicina - Porto Alegre - RS - Brasil; 3 - Pontifícia Universidade Católica do Rio Grande do Sul - PUCRS, Escola de Medicina - Porto Alegre - RS - Brasil

**Keywords:** General Surgery, Medical Oncology, Surgical Oncology, Catheters, Vascular Access Devices, Cirurgia Geral, Oncologia, Cateteres, Dispositivos de Acesso Vascular, Oncologia Cirúrgica

## Abstract

**Objective:**

to demonstrate that the use of the portocath implantation technique without tunneling the catheter is not associated with a higher rate of complications in the short or long term. In addition, we aim to improve the implantation technique of the portocath device, with the presentation of a step-by-step guide for surgeons in training.

**Methods:**

this is a retrospective descriptive study, with analytical components. Data were analyzed using information extracted from electronic medical records linked to the National Health Care procedure code between the years 2019-2020.

**Results:**

none of the 94 procedures resulted in complications on the day they were performed. Complications were recorded seven days after the procedure in only two patients (2.13%). Intraoperative radioscopy had been performed in both cases. Thirty days afters the procedure, complications were observed in two patients among the remaining 92 (2.17%), both undergoing catheter implantation without tunneling. There were no complications in the six months after portocath implantation in 57.4% of patients and there is no information about the other 42.6%.

**Conclusion:**

the portocath insertion technique without tunneling is a safe outpatient procedure, with a low risk of complications, and can be adopted to shorten procedure time and patient discomfort, without functional or safety impairments. There was no association of not tunneling the catheter, laterality of the punctured vein and performing radioscopy in the transoperative period with the rate of complications.

## RESUMO

**Objetivo:**

demonstrar que a utilização da técnica de implantação do portocath sem a etapa de tunelização não está associado a maior taxa de complicações a curto ou longo prazo. Além disso, almeja-se aperfeiçoar a técnica da implementação do dispositivo portocath, com a apresentação de um guia passo a passo para conduzir os cirurgiões em formação.

**Métodos:**

trata-se de um estudo retrospectivo descritivo, com componentes analíticos. Os dados foram analisados por meio das informações extraídas dos prontuários eletrônicos vinculados código do procedimento SUS entre 2019-2020.

**Resultados:**

nenhum dos 94 procedimentos culminou em complicações no dia de sua realização. Foram registradas complicações após sete dias do procedimento em apenas dois pacientes (2,13%). A radioscopia intraoperatória havia sido realizada em ambos os casos. Após 30 dias do procedimento, foram observadas complicações em dois pacientes entre os 92 restantes (2,17%), ambos submetidos ao implante do cateter sem tunelização. Não houve complicações seis meses após o implante do portocath em 57,4% dos pacientes e não há informação acerca dos outros 42,6%.

**Conclusão:**

a técnica inserção do portocath sem tunelização é um procedimento ambulatorial seguro, de baixo risco de complicação, podendo ser adotada como forma de abreviar tempo de procedimento e desconforto ao paciente, sem prejuízos funcionais ou de segurança. Não houve associação entre não tunelizar o cateter, lateralidade da veia puncionada e realização de radioscopia no transoperatório com a taxa de complicações.

## INTRODUCTION

The blood system has been a subject of study since ancient Greece, with descriptions of humoral theories by Hippocrates and Gales, substantially evolving with the description of the physiology of blood vessels by William Harvey in the 17^th^ century. Only in the 20^th^ century, in a post-war context, the French military surgeon Aubaniac described the first insertion of non-implantable catheters into the subclavian vein, in 1952^1^. The following year, the Swedish radiologist Seldinger published the innovative technique of insertion of intravascular catheters by puncture with the aid of a guide wire, a technique which bears his name to this day^1^ ^-^ ^3^. 

The first fully implantable catheter was described in 1972 by Belin and colleagues and was used for total parenteral nutrition. About 10 years later, the use of these devices was extended to other purposes, including the administration of chemotherapy in the treatment of cancer (one of the biggest public health problems), where it was proved to be safe and comfortable. Furthermore, the catheter gives patients freedom to perform their daily activities and improves their quality of life, without the need for multiple punctures performed for each chemotherapy^1^ ^,^ ^3^ ^-^ ^6^. 

The portocath, a type of fully implantable catheter used for chemotherapy, has a diameter smaller than 10 Fr. For installation, a central vein (internal jugular or subclavian, with or without direct ultrasound-guided visualization of the vessel) is punctured and the flexible catheter - inserted using the Seldinger technique - is connected to the reservoir housed over the muscular fascia at the site designated for making the pocket, in general the infraclavicular region^5^ ^-^ ^6^. The literature advocates that subcutaneous tunneling between the puncture site and the reservoir pocket provides greater durability to the long-term catheter, as it is a protective factor against infections^1^ ^,^ ^3^. 

Insertion of a portocath is contraindicated in the presence of active infection, severe coagulation changes, and lesions in the region where the procedure would be performed (cervico-thoracic)^3^. Among the risks of the technique, the most feared early complications are pneumo and hemothorax, with late complications related to infection and thrombosis at the procedure site^4^. 

This study aims to review and present data regarding the implementation of portocath between 2019-2020 in the General and Digestive System Surgery Service of a high-complexity hospital and to analyze the data according to the questions: Is the procedure without tunneling associated with a higher rate of complications? Is the laterality of the vein puncture associated with complications in the procedure? Is the use of radioscopy associated with decreased complication rates? In addition, the aim is to present, in an illustrative way, the surgical technique performed in the service.

## METHODS

This is a retrospective, descriptive study with analytical components. Data were analyzed using information extracted from electronic medical records linked to the SUS procedure 04.06.02.007-8 (implantation of semi or fully implantable long-term catheter) between 2019-2020. Procedures that did not correspond to the implementation of a portocath were excluded, despite the presence of the code.

 The variables studied were patient’s age at the time of operation, sex, type of neoplasm, chosen vein, punctured side, intraoperative radioscopy, control radiography on the day, as well as complications on the day, one week, one month, and six months after the procedure. Furthermore, an original step-by-step guide was described and illustrated, demonstrating the technique used in the surgical service of insertion of a portocath without tunneling. 

Numerical variables are presented as mean and standard deviation or median and quartiles (25-75%). Categorical variables are presented in absolute and relative frequencies. The chi-square (2x2) or Fisher’s exact tests were used, in addition to the chi-square (rxc), to assess the occurrence of complications on the day of implantation, and 30 and 60 days after the procedure related to non-tunneling, laterality, and the use of intraoperative radioscopy. Significance was set at 5%. All analyses were performed using IBM-SPSS version 27 (IBM SPSS^®^, Armonk, NY, USA).

This study follows the conditions established in Resolution nº 466/12 of the National Health Council (CNS), and was approved by the Ethics in Research Committee of the Institution (opinion number 4,839,930) and registered online at Plataforma Brasil (CAAE: 47237421.2.0000.5336). 

## RESULTS

Of the 94 patients included, 55.3% were women. The median age was 59 years (50-67%), ranging from 19 to 81. The three most frequently found tumors in patients undergoing portocath implants were colorectal (41.1%), breast (16%), and pancreas (10.6%), followed by gastric (6.4%), esophageal (5.3%), lymphomas (4.3%), and oropharynx (3.2%), as showned in Table 1. 

**Table 1:** Cancer sites.

Site	n	%
Colorectal	39	41.1
Breast	15	16.0
Pancreas	10	10.6
Gastric	6	6.4
Esophageal	5	5.3
Lymphomas	4	4.3
Oropharynx	3	3.2
Cholangiocarcinoma	2	2.1
Soft tissue	2	2.1
Nonmelanoma skin cancer	1	1.1
Duodenal	1	1.1
Pelvic	1	1.1
Adrenal	1	1.1
Melanoma	1	1.1
Lung	1	1.1

Table 2 shows the characteristics of the procedures. Insertion of the catheter was most common in the subclavian vein (94.7%), predominantly on the right side (85.1%). The tunneling process was performed in a minority of patients (11.7%). Intraoperative radioscopy was performed in 89.4% of the procedures, was not performed in 4.3% of the cases, and there was no information about it in 6.4% of the analyzed records. A chest X-ray to verify the correct implantation of the device was carried out after 89.4% of the procedures, not performed after 2.1% of them, and there was no information about it in 8.5% of the records.

**Table 2:** Procedures’ characteristics.

	n	%
Site		
Subclavian vein	89	94.7
Internal jugular vein	05	5.3
Side		
Right	80	85.1
Left	14	14.9
Tunneling		
Performed	11	11.7
Not performed	83	88.3
Intraoperative radioscopy		
Performed	84	89.4
Not performed	4	4.3
No data	6	6.4
Postoperative X-ray		
Performed	84	89.4
Not performed	2	2.1
No data	8	8.5

None of the 94 procedures resulted in complications on the day of the procedure. Complications were recorded seven days after the implantation in only two patients (2.13%), one of whom had undergone implantation with tunneling in the left internal jugular vein (malfunction) and the other without tunneling in the right subclavian vein (infection in the puncture site) (p=0.20). Intraoperative radioscopy had been performed in both cases. In total, 69% of patients had no complications after this period (one week) and there is no information on the remaining 28.7% (p=0.91). 

Thirty days after the procedure, complications were observed in two patients out of 92 (2.17%); both had undergone catheter implantation without tunneling; one had been punctured on the right (signs of infection) and the other on the left (non-functioning) (p=0.34). Intraoperative radioscopy had been performed in both cases.

Considering the association between laterality of the puncture site and complications (both on the day and after 30 and 60 days), we also found no significant differences between these variables (p=0.99; p=0.87, and p=0.54). The same result was found when observing intraoperative radioscopy and the occurrence of complications (p=0.98; p=0.24 and p=0.74). Table 3 shows the analysis of outcome of all performed procedures.

**Table 3:** Surgical Outcomes.

	n	%
Complications on the first day		
No	93	100
Complications in 7 days		
Yes	2	2.1
No	65	69.1
No data	27	28.7
Complications in 30 days		
Yes	2	2.1
No	75	79.8
No data	17	18.1
Complications in 6 months		
No	54	57.4
No data	40	42.6
Portocath removed in 6 months		
Yes	4	4.3
No	63	67.0
No data	27	28.7

### Portocath step-by-step implantation guide

#### Preoperative evaluation

This is an elective procedure. Before preparation, it is essential to determine the indication and rule out contraindications, such as current sepsis or coagulopathies. If contraindicated, wait for clinical optimization^3^. The implantation of a portocath can be performed with local anesthesia combined with sedation or with general anesthesia - depending on the clinical conditions and resources available at the site. Fasting is required on the day of the procedure, according to current guidelines in Brazil. Antibiotic prophylaxis is not required.

#### Step 1: Preparation

Separate the materials that will be used (a puncture tray, the portocath kit, and the sutures that will be used for catheter fixation and subcutaneous and skin closure). Wear a lead apron in the cervical and thoracic/abdominal region for protection from the radioscopy that will be performed. Wash hands according to the institutional protocol, put on a coat and sterile gloves. The patient will be sedated or under intravenous general anesthesia, in the supine position, in Trendelenburg, with the face turned to the contralateral side of the puncture site; the arms must be kept closed and at the side. Perform asepsis, and antisepsis with alcoholic chlorhexidine at the puncture site, and proceed with the placement of sterile drapes in the surroundings.

#### Step 2: Subclavian vein puncture

Place two fingers parallel and below the mid-distal third of the clavicle and, with a syringe attached to the needle, infiltrate this site with anesthetic (the solution used in this institution is a mixture of 20mL 2% lidocaine with vasoconstrictor, 20mL 7.5% ropivacaine, and 20ml of 0.9% saline solution). Anesthetize the site to be punctured towards the clavicle and immediately below the puncture site, where the pocket for the reservoir will be made. Then, with the puncture needle of the kit connected to a syringe with 3mL of 0.9% SF, puncture in a cranial direction and to the furcula, at an angle of 30 degrees. Be careful not to increase the angle of the needle due to the risk of inadvertent puncture of adjacent structures. With the subclavian vein punctured, pass the guide wire, remove the needle (at this time, cardiac arrhythmia can be seen on the monitor - which corresponds to the presence of the guide wire in the right atrium - if present, pull the guide wire until the rhythm normalizes). Secure the guide wire in the field with tweezers. Perform radioscopy to make sure the guidewire is correctly positioned.

#### Step 3: Making the pocket for the reservoir

After radioscopy and confirmation of the position, create the pocket for the reservoir parallel to the punctured guide wire, in the infraclavicular region. Use the puncture site as the median between the distal points to be sectioned. You can use the reservoir to measure the ends, leaving a mark on the skin with toothed forceps. With the scalpel, make the longitudinal incision in the skin from end to end. With the electrocautery - be very careful not to let the device touch the guide wire, resulting in an electrical discharge to the patient - follow the line previously cut in coagulation mode, opening the subcutaneous tissue and the muscular fascia in a caudal direction, undoing the fibrotic beams, alternating between cautery and blunt finger dissection. This procedure can be performed only with the surgeon or with an assistant, who will help expose the field and take care of hemostasis. Do this step firmly but gently. Insert a moistened gauze in the place where the reservoir will lay - if it all fits inside the space, this step is complete.

#### Step 4: Inserting the Catheter

Returning the attention to the guidewire, pass the introducer through the middle of it to the end, and then remove the middle. With the catheter cut to 30cm, insert it about 20cm inside the guide wire in the introducer, while opening the sides of the introducer. Afterwards, the guide wire can be removed with or without radioscopy. Cut 10cm of the catheter, pass the plastic piece with the black line on one side facing cranially to the catheter, connect the middle of the catheter to the reservoir, and attach it to the plastic piece next to the reservoir until it clicks. The device is mounted. With the needle that comes in the kit, puncture the reservoir, and make a brief blood flow and reflux test. Inject 3mL of 0.9% saline to clean the base of the reservoir. Afterwards, repeat the procedure with 4mL of diluted heparin (in a 10mL syringe with 9mL of 0.9% saline and 1mL of 5,000 IU/mL heparin). Review hemostasis before going to the next step.

#### Step 5: Reservoir fixation and synthesis

In this last step, the reservoir will be fixed to the muscle fascia so there is no risk of rotation or kink, rendering the use of the device unfeasible. Secure one side of the reservoir, then the other with Vycril 2-0. It is still possible to make an X stitch to anchor the reservoir-catheter connection in the subcutaneous tissue. Then bring the subcutaneous tissue together with simple Vycril 3-0 stitches. Finally, finish with single stitch Mononylon 4-0 or with intradermal Monocryl 3-0, depending on preference. Hypafix can be used on the surgical wound or a bandage with gauze and micropore tape.

#### Step 6: Final Step

**Figure 1:**
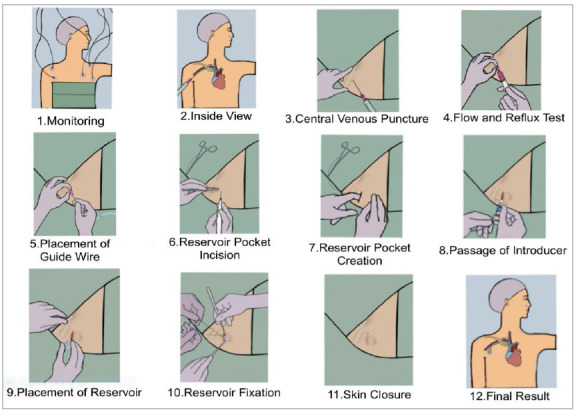
Step-by-step sequence of the puncture in the right subclavian vein and insertion of the right infraclavicular Portocath, without tunneling the catheter.

Request a control radiograph of the procedure, to be performed in the recovery room. Patients undergoing this procedure may be discharged on the same day, after recovery from sedation/anesthesia and when meeting other hospital discharge criteria. Figure 1 illustrates the step-by-step sequence in the right subclavian vein and insertion of the infraclavicular portocath on the right, without tunneling the catheter.

## DISCUSSION

In the present study, the questions asked were answered by the lack of association between complication rates and non tunneling, the laterality of the punctured vein, and the performance of intraoperative radioscopy. 

The possible complications of this procedure are divided into immediate and long-term complications, the most feared being hemothorax and pneumothorax. It is noteworthy that we did not observe either of these in the current study. In the long term, they were described as skin necrosis, inflammation, hematoma, chronic pain, and malfunction of the device were described, so removal was necessary (2.13% within 7 days and 2.17% within 30 days after the procedure). In a study carried out in South Korea, of the 397 patients analyzed after subclavian or internal jugular puncture, 8.3% presented none of the above complications, except hemo or pneumothorax, which agrees with our findings^4^. In another Canadian study on central venous catheter insertion with 6,875 patients, 23 (0.33%, 95% CI 0.22 0.5) had pneumothorax and 131 (1.91% 95% CI 1.61 2.26), inadequate placement of the catheter^7^. 

In the present study, the vein puncture was performed in most cases on the right side (85.1%). Although we found no positive association between left laterality and complications, in the same study in Canada, puncture of the left subclavian vein was described as the site with the highest risk of pneumothorax (OR = 6.69, 95% CI 2.45 18.28, p<0.001)^7^.

In our study, intraoperative radioscopy was not associated with a negative or positive outcome in terms of complications. It is believed that its importance is only to assess whether the guide wire is well positioned during the procedure, so that there are no surprises, such as poor positioning of the catheter, a rare fact described in the literature^8^ ^-^ ^10^. 

In an Italian study, 403 patients underwent insertion of a portocath using anatomical landmarks to puncture the internal jugular vein or using ultrasound to guide the puncture of the subclavian vein. No differences were found in terms of complication rates in relation to the different puncture sites, and complication rates were lower when compared with the Canadian study. That publication advocates the use of ultrasonography for puncture of the subclavian vein, which is not part of the routine of the high complexity institution where this review was carried out^5^. 

As for the limitations of this study, the retrospective nature, the fact that it was limited to 94 cases, and the absence of sample calculations stand out. 

Due to the high prevalence of cancer and the growing necessity for implantation of portocaths, the need to study this procedure’s technique is justified. Although the results are not statistically significant, they are important to foster discussion and to lay the foundation for further studies on the subject. Determination of the most appropriate insertion technique will minimize the risk of complications in the short, medium, and long term.

## CONCLUSION

The technique of portocath insertion without tunneling is a safe outpatient procedure, with a low risk of complications, and can be adopted as a way of shortening the procedure time and patient discomfort, without functional or safety impairments. There was no association of non-tunneling, laterality of the punctured vein, and performing perioperative radioscopy with the rate of complications.
